# 2597. Postoperative Pneumonia Characteristics following Pulmonary Thromboendarterectomy (PTE)

**DOI:** 10.1093/ofid/ofad500.2212

**Published:** 2023-11-27

**Authors:** Shardul N Rathod, Pamela Ropski, Jakub Glowala, Charles M Quinn, Alyssa Stamper, Maureen K Bolon, Daniel Schimmel, S Chris C Malaisrie, Ruben Mylvaganam, Mike J Cuttica

**Affiliations:** Northwestern Memorial Hospital, Chicago, Illinois; Northwestern University, McGaw Medical Center, Chicago, Illinois; Northwestern Memorial Hospital, Chicago, Illinois; Northwestern University, Chicago, Illinois; Northwestern University, Chicago, Illinois; Northwestern University Feinberg School of Medicine, Chicago, Illinois; Northwestern Medicine, Chicago, Illinois; Northwestern, Chicago, Illinois; Northwestern University Feinberg School of Medicine, Chicago, Illinois; Northwestern University: Feinberg School of Medicine, Chicago, Illinois

## Abstract

**Background:**

Pulmonary thromboendarterectomy (PTE) surgery, the gold standard for treatment of chronic thromboembolic pulmonary hypertension (CTEPH), is complicated by reperfusion lung injury (RPLI) in anywhere from 10-40% of cases. Postoperative pneumonia (PNA) following PTE is not well described, but its rates of 17-35% may be higher than expected compared to other cardiothoracic surgeries (5-6%, STS ACSD). Unique features of this highly specialized surgery may predispose patients to increased risk of postoperative PNA. This study aims to describe the incidence of suspected/confirmed post-PTE PNA and assess diagnostic/clinical characteristics and outcomes.

**Methods:**

All patients (n=75) were treated at a single-center CTEPH referral center and underwent PTE between 2016 and 2022. A retrospective chart review was conducted to assess clinical symptoms and signs of RPLI and/or PNA within 7 days of PTE. Microbiologic data was collected via bronchoalveolar lavage (BAL) within 7 days of PTE. Cohorts with microbiologic evidence of PNA and those with RPLI were compared.

**Results:**

Demographics are listed in Table 1. Of the 75 patients who underwent PTE, 28% were documented to have RPLI (mean of 2 days post-PTE) and 21% with PNA (mean of 4 days post-PTE). Of those with RPLI, 43% underwent bronchoscopy with 56% positivity 4 days post-PTE on average (Table 2). Patients with post-PTE PNA were treated with antibiotics for a mean of 6.2 days with *Staphylococcus aureus* (40%) and *Pseudomonas aeruginosa* (40%) being the most commonly isolated causative organisms. Patients with post-PTE PNA had longer intensive care unit (ICU) length of stay (LOS) (16.4 vs. 5.6 days, p=0.044) and duration of mechanical ventilation (MV) (7.4 days vs. 1.5 days, p=0.033) along with higher white blood cell counts on postoperative day one (18.3 vs. 14.8 x10^3^µL, p=0.035) (Figure 1).

**Figure:**
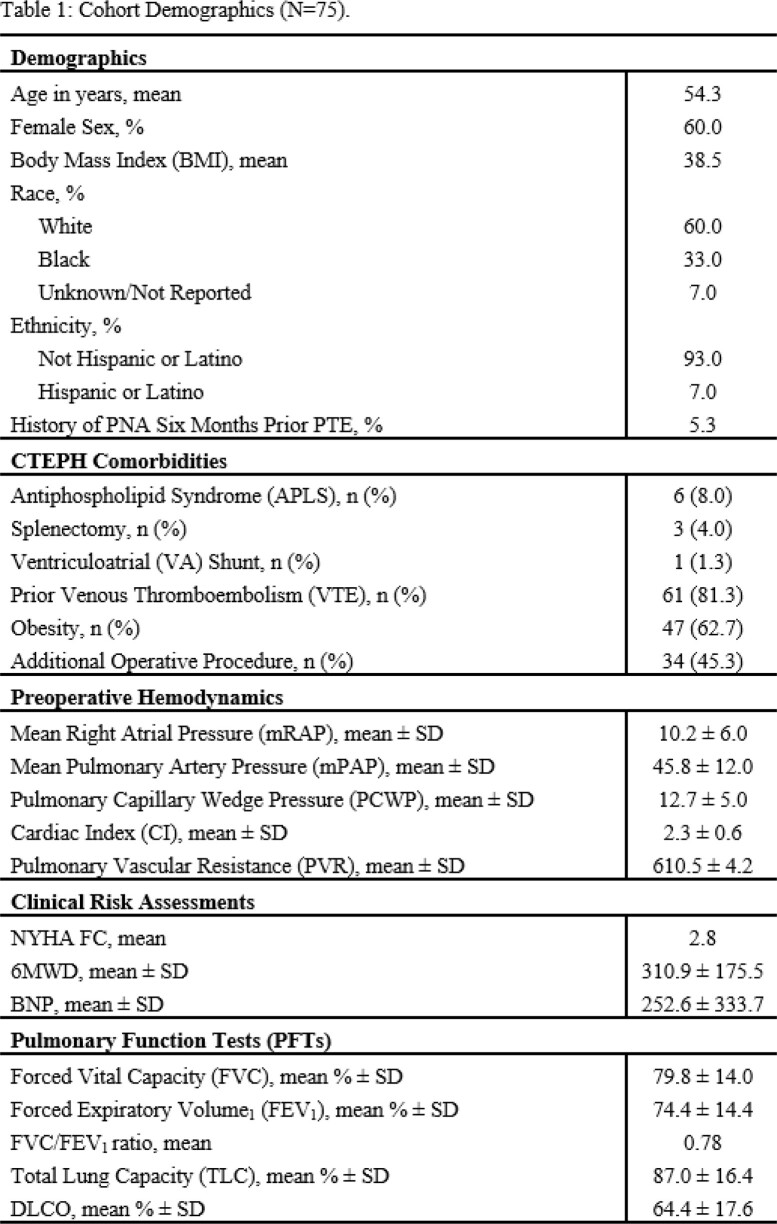

**Figure:**
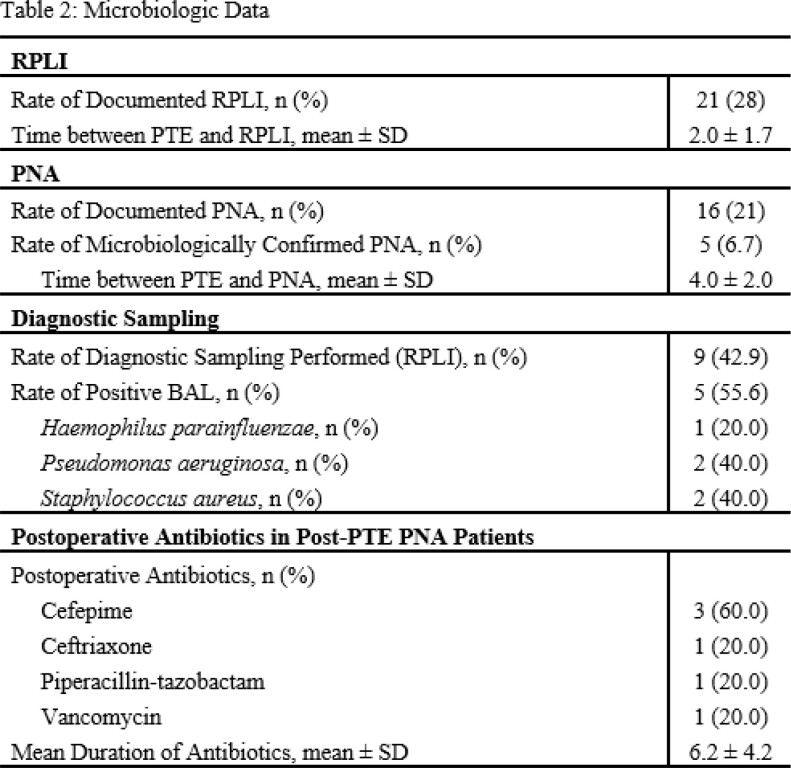

**Figure:**
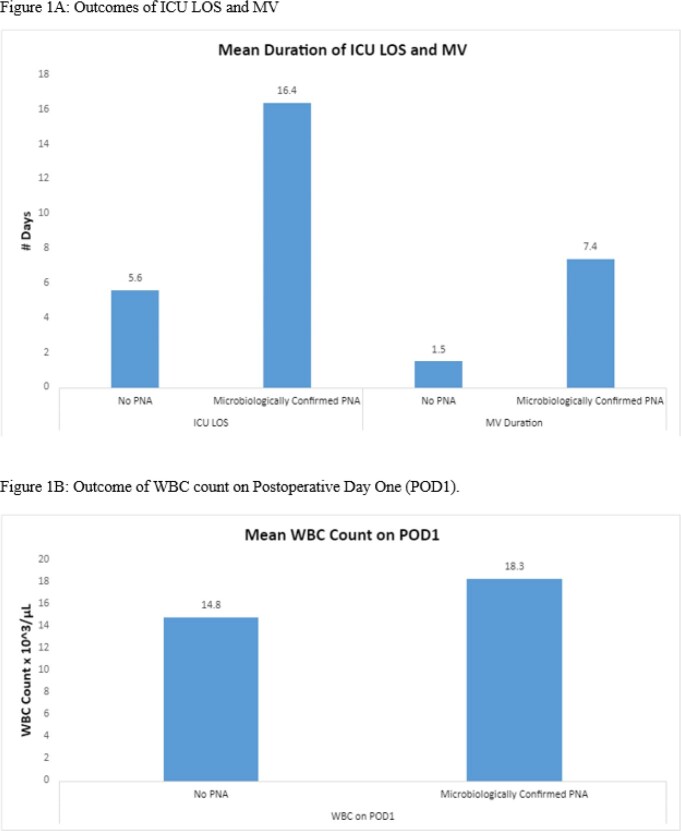

**Conclusion:**

The cohort demonstrated 28% of patients with RPLI and 21% of patients with PNA. Of those with RPLI who underwent bronchoscopy, over half the patients had positive cultures. Given that PNA is correlated with prolonged ICU LOS and duration of MV, clinicians may consider more systemic evaluation for PNA when considering RPLI, possibly warranting a change in management in these critically ill patients.

**Disclosures:**

**Daniel Schimmel, MD**, Inari: Advisor/Consultant|Inari: Grant/Research Support|Inari: Honoraria|Penumbra: Advisor/Consultant|Penumbra: Honoraria **Mike J. Cuttica, MD, MS**, Bayer: Advisor/Consultant|Bayer: Grant/Research Support|Bayer: Honoraria

